# Lecture-Based and Hands-On Ergonomic Training for Junior Obstetrics and Gynecology Residents: A Quality Improvement Pilot Study

**DOI:** 10.7759/cureus.106484

**Published:** 2026-04-05

**Authors:** Lauren Clarfield, Laura Diamond, Jennifer Diamond, Eliane M Shore, Alysha Nensi

**Affiliations:** 1 Obstetrics and Gynecology, University of Toronto, Toronto, CAN; 2 Occupational Science and Occupational Therapy, University of Toronto, Toronto, CAN; 3 Obstetrics and Gynecology, St. Michael's Hospital, Toronto, CAN

**Keywords:** curriculum, education, ergonomics, laparoscopy, medical, wellness, work-related injury

## Abstract

Objective

Determine the efficacy, feasibility, and attitudes surrounding lecture-based and hands-on ergonomic training in junior obstetrics and gynecology residents.

Methods

A mixed-methods prospective pre-post study evaluating the efficacy of a new didactic lecture and one-on-one hands-on ergonomics workshop for junior residents. Paired sample t-test was used to compare pre- and post- survey scores. Posture was evaluated using the Rapid Upper Limb Assessment (RULA) tool.

Results

Of the nine participating residents, the average age was 27.33 years, 8/9 (88.9%) identified as cis-female, and 8/9 (88.9%) were in post-graduate year one. Zero (0%) and 4/9 (44.4%) reported exposure to any formal or informal ergonomics education in residency thus far, respectively.

When comparing pre-and post-intervention survey data, residents identified improved knowledge of surgical ergonomics, increased awareness of available guidelines, improved perception of the importance of ergonomics training in residency, and stronger belief that time should be allocated to surgical ergonomics training in residency (p<0.001, p=0.020, p=0.034, and p=0.011, respectively).

On average, RULA scores improved following intervention (4.00±0.71 vs 3.56±0.53, p=0.052), including 1 point improvement for the majority of participants (n=5/9, 55.6%), no change for three residents (33.3%), and only one subject with worsening posture (11.1%), these results were not statistically significant.

Conclusions

Lecture-based and hands-on ergonomic training was a feasible and acceptable strategy to improve posture, improve awareness and knowledge of surgical ergonomics, and change attitudes regarding the importance of surgical ergonomics education during residency. Future studies should expand ergonomics education to all residency years and review whether this formal curriculum confers long-lasting change in a learner’s ergonomics.

## Introduction

Work-related musculoskeletal disorders (WMSDs), defined by the Centre for Disease Control as “an injury or disorder in which the work environment and performance of work contribute significantly to the condition; and/or the condition is made worse or persists longer due to work conditions,” are highly prevalent among surgeons [1]. Specifically, a review of the literature published in 2018 identified the incidence of WMSDs associated with operating to be as high as 66-94% for open surgery, 73-100% for conventional laparoscopy, 54-87% for vaginal surgery, and 23-80% for robotic-assisted laparoscopic surgery [2]. WMSDs are typically chronic, insidious injuries with significant physical, psychosocial, and economic impacts for surgeons, patients, and the greater healthcare system. For example, approximately one-third of gynecologic oncologists who reported physical discomfort related to performing minimally invasive surgery (MIS) required treatment for their injuries, ranging from physiotherapy (59%) to medical management (28%) and surgery (13%) [3]. When looking at surgical trainees, WMSDs among surgical residents were associated with significantly lower work satisfaction and increased negative behaviour towards others [4]. At the health systems level, WMSDs result in modified practice behaviours, including increased time off work (22-27%), modified or reduced caseloads (35-42.5%), and early retirement (9.2%) [5-7].

Despite several guidelines outlining optimal ergonomics for laparoscopic surgery [2, 8, 9], awareness and implementation of these guidelines are reported as extremely limited [10]. In addition, although WMSDs are reported to start in residency or fellowship, formal and informal education on ergonomics during training is rare [10-20] - estimated in a large-scale survey of surgical program directors to be present in only 1.5% and 25.4% of programs, respectively [18].

While several papers identify residency as an opportune time for ergonomics training [9, 19-21], there is a paucity of research outlining effective educational interventions [11-17]. The objective of this study is to evaluate the feasibility, acceptability, and attitudes surrounding a novel two-part ergonomics training intervention for junior obstetrics and gynecology residents, consisting of [i] a didactic lecture and [ii] individual postural feedback training during a laparoscopic skills simulation on ergonomic positioning. We hope the results from this pilot study lay the foundation for future curricula adaptation and further research to optimize surgical ergonomics teachings in surgical residency programs.

This article was previously presented as a meeting abstract at the 2024 American Association of Gynecologic Laparoscopy Global Congress on MIGS, the 2024 Canadian Society for the Advancement of Gynecologic Excellence CanSAGE Annual Conference, and the 2024 University of Toronto OB GYN Research Day.

## Materials and methods

Project planning

Research ethics board approval was obtained through the University of Toronto (UofT) office for Human Research (Protocol number 00044315).

Written informed consent for participation in the study was obtained from resident trainees. The interventions of this study, [i] didactic lecture and [ii] individual haptic feedback, were integrated into an existing six-session laparoscopic skills course for first-year obstetrics and gynecology residents in the UofT program from January to May. This course is purely for resident education and is not evaluated. Residents were informed that non-participation or withdrawal from participation would have no impact on training received or impact any relationship with faculty, the program, or the hospitals at which they work. 

An a priori sample size calculation was not performed because this study was designed as a pilot feasibility project to inform potential curriculum change within a single residency program. All eligible junior residents were invited to participate, and the study therefore used a convenience sample of the entire target population (n=9). Given the small, fixed size of the residency cohort, recruitment was not based on statistical power considerations but rather on comprehensive program inclusion to assess feasibility, acceptability, and preliminary efficacy.

Intervention and data collection

Consenting participants received an envelope containing a demographic survey and a randomly assigned study ID number. The demographic survey assessed resident characteristics, preexisting musculoskeletal symptoms and pain, and current attitudes and knowledge level regarding surgical ergonomics. 

As part of the training session, residents were tasked with learning a peg transfer exercise using a laparoscopic box trainer. They were challenged to pick up six discs from the left side of the array of pegs, transfer these discs between their dominant and non-dominant laparoscopic graspers, and place each disc on the right side of the peg array. The individuals then performed the same task backwards, transferring the discs back to their original pegs. Participants were then invited into a private simulator space with minimal distractions and no peer-to-peer audience, where they were filmed for five minutes. The video file was stored with a randomized code. 

All present residents, regardless of participation, then participated in a one-hour virtual lecture from an American leader in surgical ergonomics and an occupational therapist from Duke University (MP). The lecture highlighted the epidemiology of surgical WSMDs and common pitfalls of ergonomics in laparoscopic surgery.

At the subsequent laparoscopic skills session, all participants were re-invited into the private simulator space. During this subsequent session, an occupational therapist (JD) provided haptic, one-on-one, postural feedback for up to five minutes as they performed the peg transfer task.

Following the haptic feedback, participants were recorded for an additional five minutes performing the laparoscopic peg transfer task. Videos were randomly coded and securely stored.

The Rapid Upper Limb Assessment (RULA) tool (Appendix 1) is a validated observational method of posture analysis to quantify risk of injury secondary to posture present for the most common observed posture and is endorsed by the Society of Surgical Ergonomics [18-20]. The full assessment worksheet is available in the original publication. A score of 1-2 represents “acceptable posture”, a score of 3-4 represents “further investigation, change may be needed”, 5-6 represents “further investigation, change soon”, and 7 represents “investigation and change soon”. The coded videos were provided to a single member of the research team (JD) with expertise in ergonomics, who analyzed each blinded video.

A post-intervention survey was distributed to participants, investigating attitudes, awareness, and knowledge surrounding surgical ergonomics. 

Data analysis 

Demographic data of participating residents is described. Paired-samples t-test was used to examine the change in knowledge, awareness, and RULA scores from pre- to post-intervention. A one-tailed (directional) test was used as the change was expected in a certain direction (increase in attitudes, knowledge, and awareness scores, and decrease in RULA scores). 

## Results

Demographic data

Of nine participants, the average age was 27.33 years, 8/9 (88.9%) identified as cis-female, and 8/9 (88.9%) were in post-graduate year one, while 1/9 (11.1%) was in postgraduate year (PGY)2 (Table 1).

**Table 1:** Demographic characteristics of study participants, n=9 PGY: postgraduate year, OR: operating room.

	Mean±SD (range)
	or Frequency (%)
Age	27.33±2.07 (24–30)
Sex	
Male	1 (11.1%)
Female	8 (88.9%)
Post-graduate year	
PGY1	8 (88.9%)
PGY2	1 (11.1%)
Height (cm)	168.89±7.66 (157–182)
Weight (lbs)	139.89±18.76 (110–170)
Glove size	
6	3 (33.3%)
6.5	4 (44.4%)
7	2 (22.2%)
Pre-existing injuries	4 (44.4%)
Lower extremity	2 (22.2%)
Spine	2 (22.2%)
Upper extremity	0 (0%)
Therapy for symptoms	7 (77.8%)
Massage	7 (77.8%)
Chiropractor	4 (44.4%)
Medical management non-opioid	3 (33.3%)
Medical management opioid	0 (0%)
Acupuncture	2 (22.2%)
Requested time off during a case or working day due to symptoms	0 (0%)
Reported symptoms to a supervisor	0 (0%)
Received formal ergonomics teaching	0 (0%)
Received informal ergonomics teaching	4 (44.4%)
Aware of a bad posture in the OR	8 (88.9%)
Feel that surgical culture prevents from adjusting the operating room ergonomics to improve posture (height of table, short breaks to flex, etc.)	7 (77.8%)

The average height was 168.89 cm±7.66, and the average weight was 139.89 lb±18.76. Three residents (33.3%) wore a size 6 glove, four (44.4%) wore a size 6.5 glove, and two (22.2%) wore a size 7 glove. If individuals wear two gloves, they were asked to identify which one they wore closest to the skin.

Nearly half of the participants (4/9, 44.4%) identified having pre-existing musculoskeletal injuries, two of which were localized to the lower extremity and two of which were spine-related. None of the participants endorsed requested time off during a case or workday due to musculoskeletal symptoms, and similarly, none reported symptoms to a supervisor. In contrast, the majority of participants (7/9, 77.8%) endorsed having sought therapy or treatment to minimize musculoskeletal symptoms or injuries, including 7/9 (77.8%) reporting prior use of massage, 4/9 (44.4%) chiropractor, 2/9 (22.2%) acupuncture, and 3/9 (33.3%) using non-opioid medical management. None of the participants reported using opioids. 

Knowledge, attitudes, and awareness surrounding surgical ergonomics

Although none of the participants reported any formal ergonomics education in residency thus far, nearly half (4/9, 44.4%) endorsed informal ergonomics training, including “supervisors advising me to straighten my back, drop my shoulders” and “staff advising me to advocate for changing the table height”. Despite limited formal education, the majority of residents (7/9, 77.8%) reported self-awareness of bad posture in the operating room. Of note, the majority (7/9, 77.8%) felt surgical culture prevented them from adjusting the operating room ergonomics to improve posture. For instance, they reported feeling unable to adjust table height as a junior learner, not wanting to interrupt a case to request a microbreak or ergonomic adjustment, and enduring a poorer ergonomic posture in order to accommodate staff surgeon height (Table 2). 

**Table 2:** Pre- and post-intervention survey responses evaluating knowledge, awareness, and attitudes toward surgical ergonomics

	Pre-intervention score Mean±SD	Post-intervention score Mean±SD	Improvement Mean±SD	Statistical test (paired-samples t-test, one-tailed)
Current understanding and/or knowledge of surgical ergonomics (1=very low, 5=very strong)	2.11±0.78	3.89±0.60	1.78±0.83	t(8)=6.40, p<0.001
Awareness of surgical ergonomics guidelines (1=no awareness, 5=very familiar)	1.89±1.05	3.00±1.41	1.11±1.41	t(8)=2.44, p=0.020
Importance of understanding of surgical ergonomics for the future career (1=not important, 5=very important)	4.67±0.50	4.89±0.33	0.22±0.44	t(8)=1.51, p=0.085
Perception of current surgical ergonomics training in residency program is (1=very poor, 5=very strong)	2.11±0.93	3.00±1.41	0.89±1.27	t(8)=2.10, p=0.034
Importance of dedicated time to surgical ergonomics education during residency is (1=not important, 5=very important)	4.22±0.67	4.89±0.33	0.67±0.71	t(8)=2.83, p=0.011

Following our two-part surgical ergonomics educational intervention, five of nine participants (55.5%) felt that this ergonomics teaching came at an appropriate time in their residency training (i.e., nearing the end of PGY1). Of the two others who answered in free text that this was not the most appropriate time, one felt earlier exposure would be more beneficial, while another individual felt training would be improved during later years when trainees are operating more consistently. When asked about the intervention itself, four of seven (57.2%) respondents thought the haptic feedback component was the most beneficial, while the remaining three (42.8%) thought the didactic lecture and hands-on component were effective in combination. None of the respondents reported that a didactic lecture alone was the most effective way for them to learn surgical ergonomics. 

When comparing pre- and post-intervention survey data on a one-to-five Likert scale, participating residents identified improved understanding and/or knowledge of surgical ergonomics and improved awareness of surgical ergonomics guidelines following our intervention (2.11±0.78 vs. 3.89±0.60, p<0.001, and 1.89±1.05 vs. 3.00±1.41, p=0.020, respectively). There was also a small, non-statistically significant improvement in the understanding of the importance of surgical ergonomics for a future career, but the pre-intervention score was high for the pre-intervention group (4.67±0.50 vs. 4.89±0.33, p=0.085), and there was improved perception of dedicated time to surgical ergonomics education during residency (4.22±0.67 vs. 4.89±0.33, p=0.011). 

Postural haptic feedback - RULA scores

**Figure 1:**
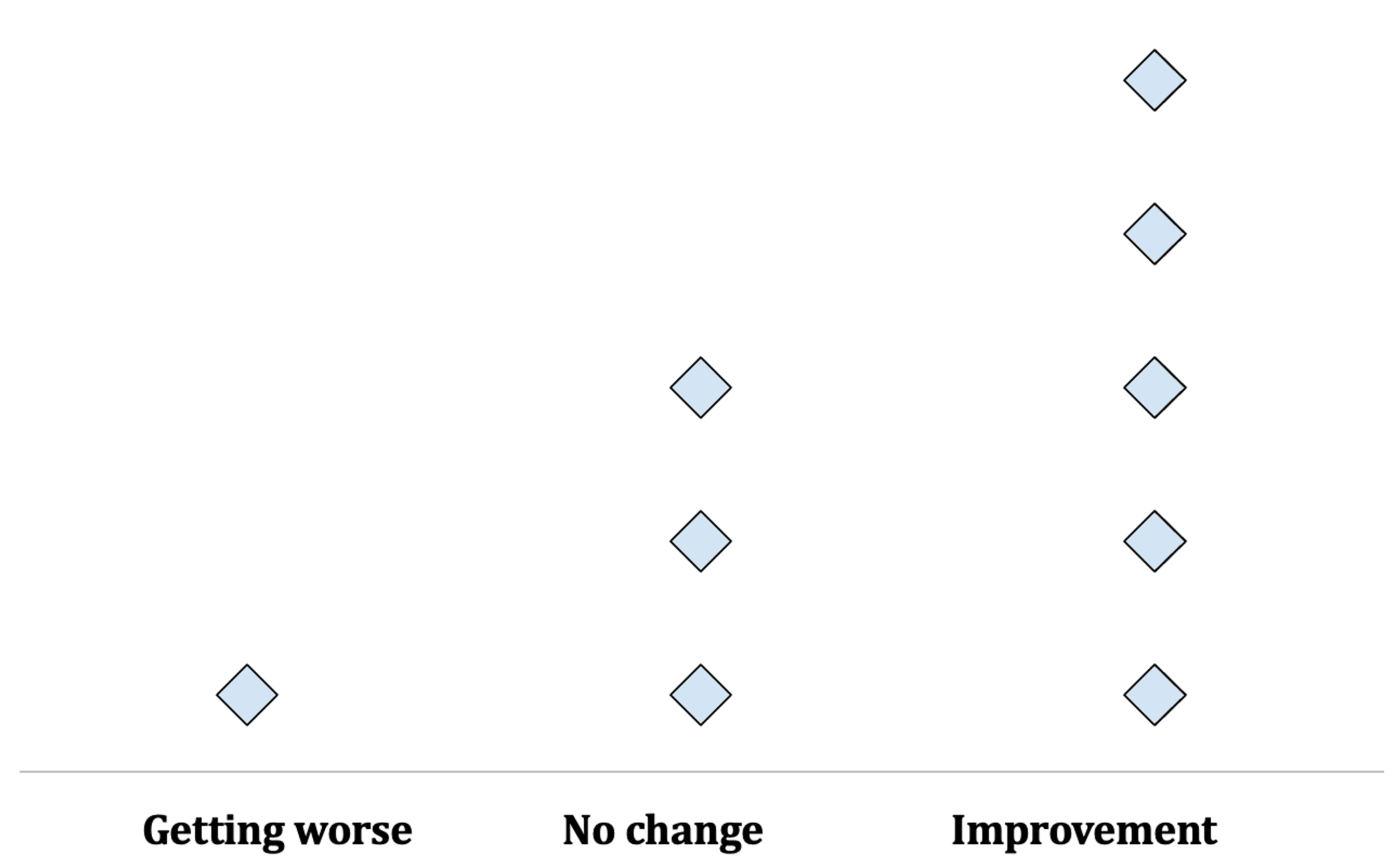
Dot plot showing change in participants’ RULA scores post-intervention

When completing the peg transfer exercise in the pre-intervention period, zero participants (0/9, 0%) were identified in the “acceptable posture” range, 7/9 (77.8%) were graded in the “further investigation, change may be needed" range, and 2/9 (22.2%) were graded in the “further investigation, change soon" range. In the post-intervention period, still, none of the participants (0/9, 0%) were identified in the “acceptable posture” range. Following the haptic feedback intervention, all residents (9/9, 100%) were graded in the “further investigation, change may be needed " range, with none graded in the “further investigation, change soon” range. RULA scores showed improvement with the majority of participants (5/9, 55.6%) demonstrating one unit improvement on the 7-point measure, three participants (33.3%) showing no change, and only one subject (11.1%) getting worse (Figure 1).

The mean difference in pre- and post-intervention scores was borderline significant, changing from 4.00±0.71 pre-intervention to 3.56±0.53 post-intervention (p=0.052) (Table 3). 

**Table 3:** Pre- and post-intervention Rapid Upper Limb Assessment (RULA) scores for residents participating in the ergonomics curriculum

	Pre-intervention score (Mean±SD)	Post-intervention score (Mean±SD)	Improvement (Mean±SD)	Statistical test [paired-samples t-test, one-tailed]
RULA score	4.00±0.71	3.56±0.53	0.44±0.73	t(8)=1.84, p=0.052

## Discussion

This pilot study identified combined didactic teaching and individual postural feedback as a feasible and effective method to improve posture during simulation in the short term for junior obstetrics and gynecology residents. This is in keeping with a 2022 pre-post intervention study carried out among general surgery residents, which indicated that lecture and personalized coaching were an effective method for ergonomics education [12], and other studies that used lecture alone [13, 17]. While just over half of our study cohort identified haptic feedback alone as the most valuable aspect of this intervention, the remainder felt that combined didactics and postural feedback was important. In contrast to other studies in the literature [13, 17], no one in our cohort deemed lecture alone to be the most effective method of teaching. From our post-intervention survey, it was clear this dual approach was not only effective at improving posture but also resulted in improved perceived knowledge and increased awareness of the importance of prioritizing ergonomics in surgery. Contrary to the extremely limited formal ergonomics teaching among U.S. surgical residencies, quoted at 1.5%[18], all participating residents felt that time dedicated to surgical ergonomics in residency was important (4.89±0.33/5), as was understanding ergonomics for future career (4.89±0.33/5). 

Even following the intervention, a large proportion of students felt there were barriers to implementing surgical ergonomics strategies in the operating room. Specifically, this study identified surgical culture as a predominant barrier. Residents identified feeling embarrassed as junior learners to ask for adjustments. They highlighted fears of being perceived as incapable of withstanding the demands of surgery and not wanting to stop or delay a case for a personal ergonomics issue. We hope future studies can focus on identifying and addressing cultural barriers to increase awareness and acceptance of surgical ergonomics. This may involve interventions beyond the simulation setting. Interventions may include the initiation of an ergonomic time-out or routine ergonomic microbreaks, targeting operating room nursing staff and surgeons [21].

Most participants (7/9, 77.8%) identified PGY1 as an appropriate year for this educational intervention. Although this study demonstrated improved surgical posture in the short term in simulation, we are unable to predict whether this intervention will make a lasting change in the natural surgical environment. Future formal surgical ergonomics interventions should include long-term follow-up and be implemented throughout various stages of training and environments to compare when and where ergonomics training will be most effective for lasting change. For example, future work should compare interventions for advanced trainees who spend more time performing laparoscopic surgery versus junior trainees who spend more time practicing obstetrics but are developing early habits in laparoscopic surgery. 

Finally, even after implementation of this dual intervention, individuals identified current knowledge of surgical ergonomics to be only at a 3.89±0.60/5 on a 5-point Likert scale and rated their perception of current surgical ergonomics training in our program at a 3.00±1.41. This highlights a student-perceived gap in ergonomics knowledge and training even beyond the curriculum proposed here. 

Limitations

There are several limitations of this study. First, the sample size was small, with only nine participants. This limits our ability to generalize our results, and there is also limited statistical power due to the lack of a control group. However, this was a pilot study to assess feasibility, acceptability, and attitudes for this style of curriculum to lay the foundation for future research. Second, although the RULA is a validated tool to predict injury risk, as endorsed by the Society of Surgical Ergonomics [22], there are several limitations of this tool including: [i] inability for the RULA to account for small changes given that a score is assigned for only the most common observed posture [i.e. a participant would receive the same score if they improved harmful abduction of their arm from 80% to 60% of time spent in posture], [ii] the RULA does not account for the dynamic nature of surgery, which sometimes requires awkward positioning, and therefore does not reward moving out of this positioning or only maintaining high-risk ergonomics postures briefly. To our knowledge, there is no better-established measure of posture to assess dynamic surgical posture for this style of intervention; however, other proposed mechanisms may include wearable devices or a survey alone [15]. The setting serves as an additional limitation in this study. Our intervention took place in a simulation environment where students were unable to adjust screen height and table height as they would in an operating room, and where stress is lower and moving to accommodate a lower risk posture would not be hindered by surgical culture nor impact surgical proficiency in a meaningful way regarding patient care. Finally, timing serves as a limitation, as the analysis also took place within the same session as the intervention, and we are therefore unable to assess the effectiveness or retention of this intervention in the long term. 

## Conclusions

This represents the first study to assess the feasibility, acceptability, and attitudes towards a formal ergonomics training intervention for obstetrics and gynecology residents. Though this is a small study, this pilot project demonstrated a need and desire for ergonomics education amongst residents and highlighted an effective and well-received two-part method of training. Next steps include the implementation of dual ergonomic training for surgical residents, including haptic feedback with a trained member of allied health work, where feasible. Future studies should assess whether this two-part format of training confers benefits beyond simulation and brings about long-lasting change for residents as they progress through their surgical careers.

## Appendices

Appendix 1 

Figure 2: Rapid Upper Limb Assessment (RULA) scoring scale.

Reprinted from Applied Ergonomics, Vol 24, McAtamney L and Corlett EN, RULA: a survey method for the investigation of work-related upper limb disorders, Pages No. 91-99, Copyright (1993), with permission from Elsevier [18]
